# Construction of an individualized brain metabolic network in patients with advanced non-small cell lung cancer by the Kullback-Leibler divergence-based similarity method: A study based on 18F-fluorodeoxyglucose positron emission tomography

**DOI:** 10.3389/fonc.2023.1098748

**Published:** 2023-03-10

**Authors:** Jie Yu, Lin Hua, Xiaoling Cao, Qingling Chen, Xinglin Zeng, Zhen Yuan, Ying Wang

**Affiliations:** ^1^ Department of Nuclear Medicine, The Fifth Affiliated Hospital of Sun Yat-sen University, Sun Yat-sen University, Zhuhai, Guangdong, China; ^2^ Faculty of Health Sciences, University of Macau, Macau, Macau SAR, China; ^3^ Centre for Cognitive and Brain Sciences, University of Macau, Macau, Macau SAR, China

**Keywords:** non-small cell lung cancer, fluorodeoxyglucose, positron emission tomography, brain metabolic network, Kullback-Leibler divergence-based similarity

## Abstract

**Background:**

Lung cancer has one of the highest mortality rates of all cancers, and non-small cell lung cancer (NSCLC) accounts for the vast majority (about 85%) of lung cancers. Psychological and cognitive abnormalities are common in cancer patients, and cancer information can affect brain function and structure through various pathways. To observe abnormal brain function in NSCLC patients, the main purpose of this study was to construct an individualized metabolic brain network of patients with advanced NSCLC using the Kullback-Leibler divergence-based similarity (KLS) method.

**Methods:**

This study included 78 patients with pathologically proven advanced NSCLC and 60 healthy individuals, brain ^18^F-FDG PET images of these individuals were collected and all patients with advanced NSCLC were followed up (>1 year) to confirm their overall survival. FDG-PET images were subjected to individual KLS metabolic network construction and Graph theoretical analysis. According to the analysis results, a predictive model was constructed by machine learning to predict the overall survival of NSLCL patients, and the correlation with the real survival was calculated.

**Results:**

Significant differences in the degree and betweenness distributions of brain network nodes between the NSCLC and control groups (*p*<0.05) were found. Compared to the normal group, patients with advanced NSCLC showed abnormal brain network connections and nodes in the temporal lobe, frontal lobe, and limbic system. The prediction model constructed using the abnormal brain network as a feature predicted the overall survival time and the actual survival time fitting with statistical significance (r=0.42, *p*=0.012).

**Conclusions:**

An individualized brain metabolic network of patients with NSCLC was constructed using the KLS method, thereby providing more clinical information to guide further clinical treatment.

## Introduction

Lung cancer is the leading cause of cancer-related death worldwide, with non-small cell lung cancer (NSCLC) accounting for the majority (approximately 85%) of all lung cancers (1–3). The nervous system, *via* neural and humoral pathways, substantially modulates processes related to cancer at the level of the tumor’s micro-and macro environments (4, 5). The nervous system also mediates the effects of psychosocial and noetic factors on cancer development (6, 7). Neurobiological perspectives on cancer pathogenesis suggest that cancer messages are transmitted to specialized brain structures through neural and humoral pathways (7–9); thus, the brain may modulate the neuroendocrine-immune system in response to tumor growth (10). Previous studies have described the potential mechanisms underlying the neuromodulation of abnormal brain activation patterns associated with lung cancer. Metabolic imaging techniques, such as magnetic resonance (MR) spectroscopy and positron emission tomography/computed tomography (PET/CT), have recorded significant changes in metabolic and functional status in the resting-state brain of patients with NSCLC (10–12).

Although numerous structural MR-based studies have used the Kullback-Leibler divergence-based similarity (KLS) method for individualized analysis, few studies have used KLS to construct individual metabolic brain networks by employing fluorodeoxyglucose (FDG)-PET imaging (13–16). In previous studies, brain MR and brain PET were used to predict NSCLC brain metastases and overall survival by machine learning and other methods (17, 18). In medicine, machine-learning techniques are widely used for the prognostic prediction of cancer (19–21).

Herein, patients diagnosed with advanced NSCLC during 2019–2021 were selected. The main objective of this study was to construct a metabolic network for each patient with NSCLC and elaborate on the possible applications of the brain metabolic network.

## Materials and methods

### Participants

The retrospective research design of this study was in line with the principles of the Declaration of Helsinki. This prospective study was approved by the Fifth Affiliated Hospital of Sun Yat-Sen University. Patients with histologically proven advanced NSCLC were enrolled from September 2017 to October 2020 and followed up until March 2022. Patients with brain tumors (primary brain tumor or metastasis), prior surgery, chemotherapy, stroke, and head trauma were excluded. The control group (n = 60) comprised those who underwent a whole-body PET/CT scan for the first time to screen for tumors and showed no evidence of malignancy in the examination. Control individuals who have a history of various types of cancer, those who do not have complete information on chemotherapy, those with primary or metastatic brain tumors detected through MRI, those who have had a stroke, head trauma, neurological diseases (such as epilepsy or dementia), or drug dependency (including alcohol, opioids, hypnotics/sedatives, cannabis, hallucinogens, or cocaine), as well as those with a history of major affective disorders (such as major depressive disorder and bipolar disorder) and psychotic spectrum disorders (including schizophrenia, delusional disorder, paranoid disorder, schizotypal disorder, and schizoaffective disorder) were excluded from the study. All control participants were further confirmed by follow-up visits for at least 12 months after PET/CT examination. The same exclusion criteria as the lung cancer group were applied to the control group.

### FDG-PET image acquisition and processing

^18^F-FDG was supplied by Guangzhou HTA Pharmaceutical Co., Ltd. Imaging was performed approximately 60 min after administration using an integrated PET/CT scanner (uMI780, United Imaging, China) from the top of the head to the upper thighs using the following parameters: 120 kV, 240mAs, and thickness of 2 mm. PET images were acquired at 2 min per bed position.

Individual FDG-PET images were processed using MATLAB (MathWorks, Natick, MA, United States) platform-based Statistical Parametric Mapping version 12 (SPM12) (https://www.fil.ion.ucl.ac.uk/spm/software/spm12/). The images were spatially normalized to a standard stereotactic template in the Montreal Neurological Institute space with linear and nonlinear 3D transformations; then, a 6 mm full-width half-maximum smoothing kernel was applied to the normalized FDG-PET images to increase the signal-to-noise ratio. To facilitate comparison across all participants, the intensity of the images was further normalized to the average whole-brain uptake. Subsequently, normalized glucose uptake values in each voxel were extracted from 90 regions of interest (ROIs; 45 for each hemisphere without the cerebellum) using an automated anatomical labelling-based atlas (22).

### Individual KLS metabolic network construction

To construct interregional network connections, we utilized the KLS method, which has been successfully used to quantify morphological connectivity between two regions (14, 15). Notably, from the perspective of information theory, KL divergence is an index that measures the difference between two probability distributions or the information lost when a probability distribution *P* is used to approximate another probability distribution *Q (* 23). Therefore, the high KLS value between the two brain regions may indicate metabolic connections subserving high inter-regional information transfer.

For each participant, glucose uptake values within each ROI were first estimated using the probability density function using kernel density estimation (24, 25) with bandwidths chosen automatically (26). This analysis was implemented using the public MATLAB code (http://www.mathworks.com/matlabcentral-/fileexchange/14034-kernel-density-estimator). The probability distribution function (PDF) was obtained from the probability density function. Subsequently, KL divergence was employed to calculate the intensity of the metabolic connection between any pair of ROIs. Formally, the KL divergence from distribution *Q* to *P* was defined as (27):


$$KL(P||Q)=\sum_{i=1}^{n}P\left(i\right)\;log\frac{P\left(i\right)}{Q\left(i\right)}$$

where *P* and *Q* represent the two PDFs of the voxel intensities in a pair of ROIs. However, *KL*(*P*  *Q*) is not equivalent to *KL*(*Q*  *P*) . Therefore, we converted KL divergence into a symmetric measurement using the following equation:


$$KL\left(P,Q\right)=\sum_{i=1}^{n}\left(P\left(i\right)\;log\frac{P\left(i\right)}{Q\left(i\right)}+Q\left(i\right)\;log\frac{Q\left(i\right)}{P\left(i\right)}\right)$$

Finally, the intensity of pairwise metabolic connectivity between the two ROIs was calculated as follows:


$$KLS\left(P,Q\right)=e^{-KL\left(P,Q\right)}$$

where *e* is a natural exponential function. Furthermore, KLS ranges from 0 to 1, where 1 represents two identical distributions. Here, the adjacency matrix of metabolic connectivity was constructed using KLS. Thus, the adjacency matrix defines pairwise metabolic connectivity, where the intensity of the connection between regions *i* and *j* is represented by the corresponding element in the adjacency matrix.

### Graph theoretical analysis

To exclude noisy elements before calculating the topological characterization, a sparsity threshold of S was applied to convert each matrix *C*_*ij*_=|*c*_*ij*_| into a weighted network:


$$W_{ij}=\left[w_{ij}\right]=\{\begin{array}{c} \left|c_{ij}\right|,\;if\;\left|c_{ij}\right|>\gamma_{threshold}\\ 0,\;others \end{array}$$

where *γ_threshold_* is the connectivity strength threshold (28). To avoid the specific selection of a threshold and address the differences in the number of edges within participants, a range sparsity threshold of S (0.02 ≤ S ≤ 0.50, interval=0.01) was applied to all metabolic network matrices.

Topological characterizations of the metabolic network were analyzed using the GRETNA toolbox (https://www.nitrc.org/projects/gretna/). For the resultant networks at each sparsity threshold, we included both global (assortativity, A_r_; modularity, Q; hierarchy, H_r_; global efficiency, E_global_; local efficiency, E_local_; clustering coefficient, C_p_; shortest path length, L_p_; synchronization, S_r_; normalized L_p_, λ; normalized C_p_, γ; small-worldness, σ) and nodal (nodal degree, D_c_; nodal betweenness, B_c_) metrics commonly used to describe the organization of metabolic networks in healthy participants and patients. To determine whether the metabolic networks were not randomly organized, the network topology was compared to 100 matched random networks that preserved the same number of nodes and edges and the same degree distribution as real metabolic networks (29, 30).

### Predicting patients’ overall survival using individual KLS metabolic network

Based on the individual metabolic connections between ROIs, a support vector machine for the regression algorithm (L2-regularized L2-loss SVR model) from the LIBLINEAR toolbox (https://www.csie.ntu.edu.tw/~cjlin/liblinear/) was trained to predict each patient’s overall survival. SVR has recently emerged as a preferred method for using imaging features to predict multiple patients’ symptoms in Alzheimer’s disease (31) and psychotic illness (32). In our study, the leave-one-out cross-validation (LOOCV) method was used with SVR model, in which individual metabolic connections’data from N-1 patients with their overall survival values were used to train the model (33). The model was then applied to the metabolic network data of the remaining patients to assess the overall survival. In current study, we constructed the predictive model based on the following steps: features selection, training and testing the SVR model with LOOCV method. Before feature selection, covariates, including age and sex, were regressed from the features and overall survival. The regression weights were applied to the remaining dataset. For features selection, to avoid over-fitting and examine whether the connections that exhibited significant difference between patients and healthy participants can track the patients’ overall survival, we firstly conducted a network connection analysis (see below: statistical analysis) between healthy participants and patients. Subsequently, for training the SVR model, significant connections in patients were chosen as features to train the model in each LOOCV. To calculate the predicted overall survival, the de-confounded features from the testing data were fed into the trained model for testing the SVR model. To assess the overall survival of all patients, the procedure was repeated N times (N=78). The estimated and observed overall rates were then compared to determine the correlation.

### Statistical analysis

A two-sample *t*-test was performed to compare the area under the curve of each network metric, including global (A_r_, Q, H_r_, E_global_, E_local_, C_p_, L_p_, S_r_, λ, γ, σ) and nodal (D_c_, B_c_) metrics, between healthy participants and patients. Network connection statistics were also conducted using a two-sample *t*-test between the connection matrices of healthy participants and patients. Age and sex were considered as control covariates. The significance criterion was *p*< 0.05, and the false discovery rate was applied for multiple comparisons.

Significant prediction of the correlation between observed and estimated overall survival was assessed using permutation testing (10,000 permutations). The percentage of permutation correlations, which was higher than the observation-prediction correlation based on the real data, was used to estimate the *p*-value of the permutation. The contributions (connection weight) of the metabolic connections were then averaged across all LOOCV folds. Finally, the connections were classified into six brain regions (frontal lobe, temporal lobe, parietal lobe, occipital lobe, central structures, insula, and cingulate gyri) (25). The weight of each brain region in overall survival estimation was calculated by adding the absolute weights of the predicted connections of the involved region.

### Visualization

**Figure 1:**
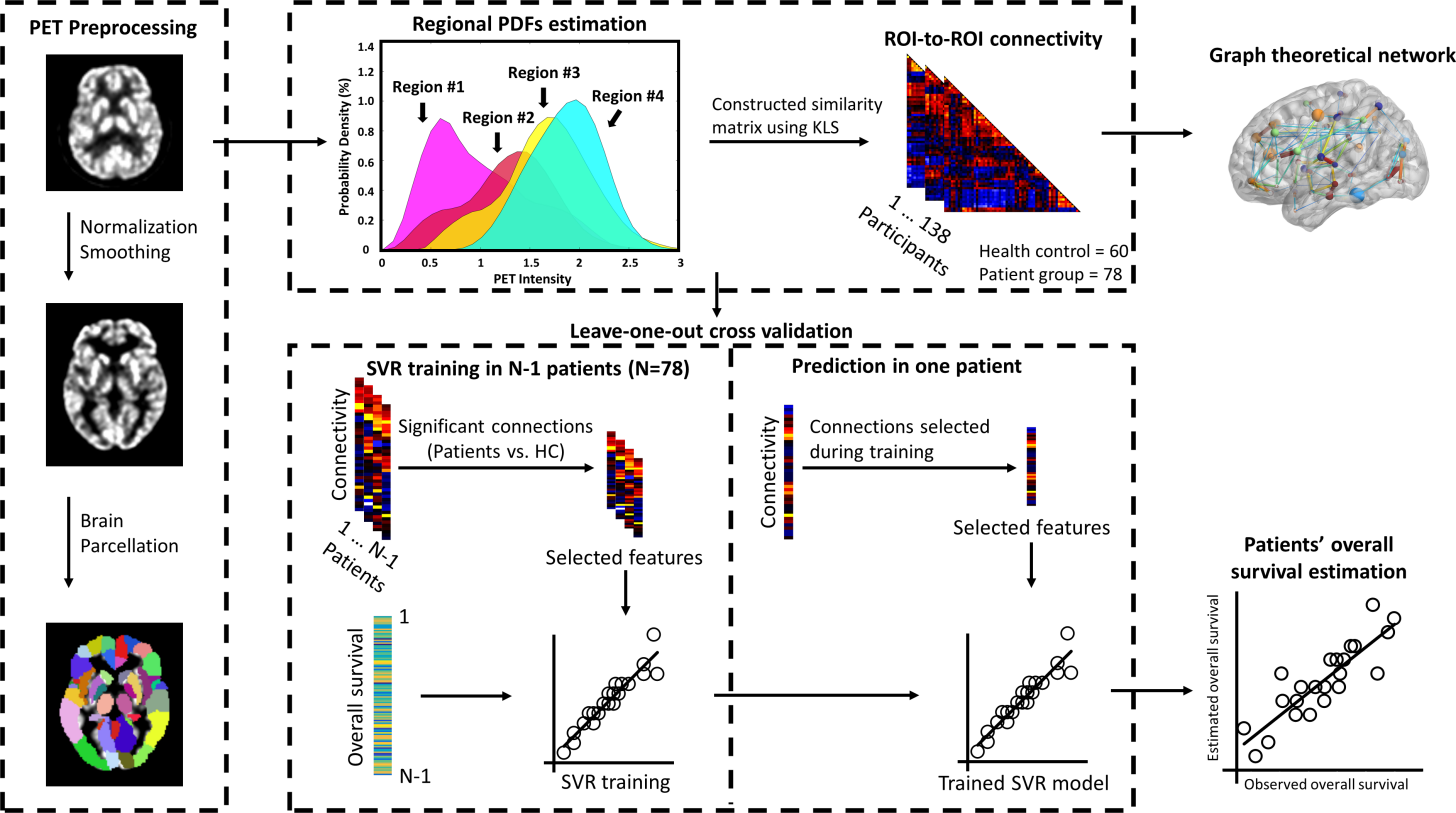
Procedure of constructing individualized brain metabolic network and predicting the overall survival in patients. Individual FDG-PET images were preprocessed using spatial normalized and smoothing. Normalized glucose uptake values in each voxel were extracted from AAL 90 regions. Then, individualized brain metabolic network was constructed using the KLS method. SVR model was trained to estimate each patient’s overall survival based on significant brain metabolic connections between healthy and patients’ groups. Data from *N*-1 patients were used to train the model and then the resulting model was applied to the data of the remaining patient to estimate the overall survival. This procedure was repeated *N* times to predict the overall survival of all patients. The correlation between the estimated and observed overall survival was then evaluated. Furthermore, brain metabolic networks between healthy and patients’ group were also systematically compared by the network’s global and local properties using the graph theoretical approach.

**Figure 2:**
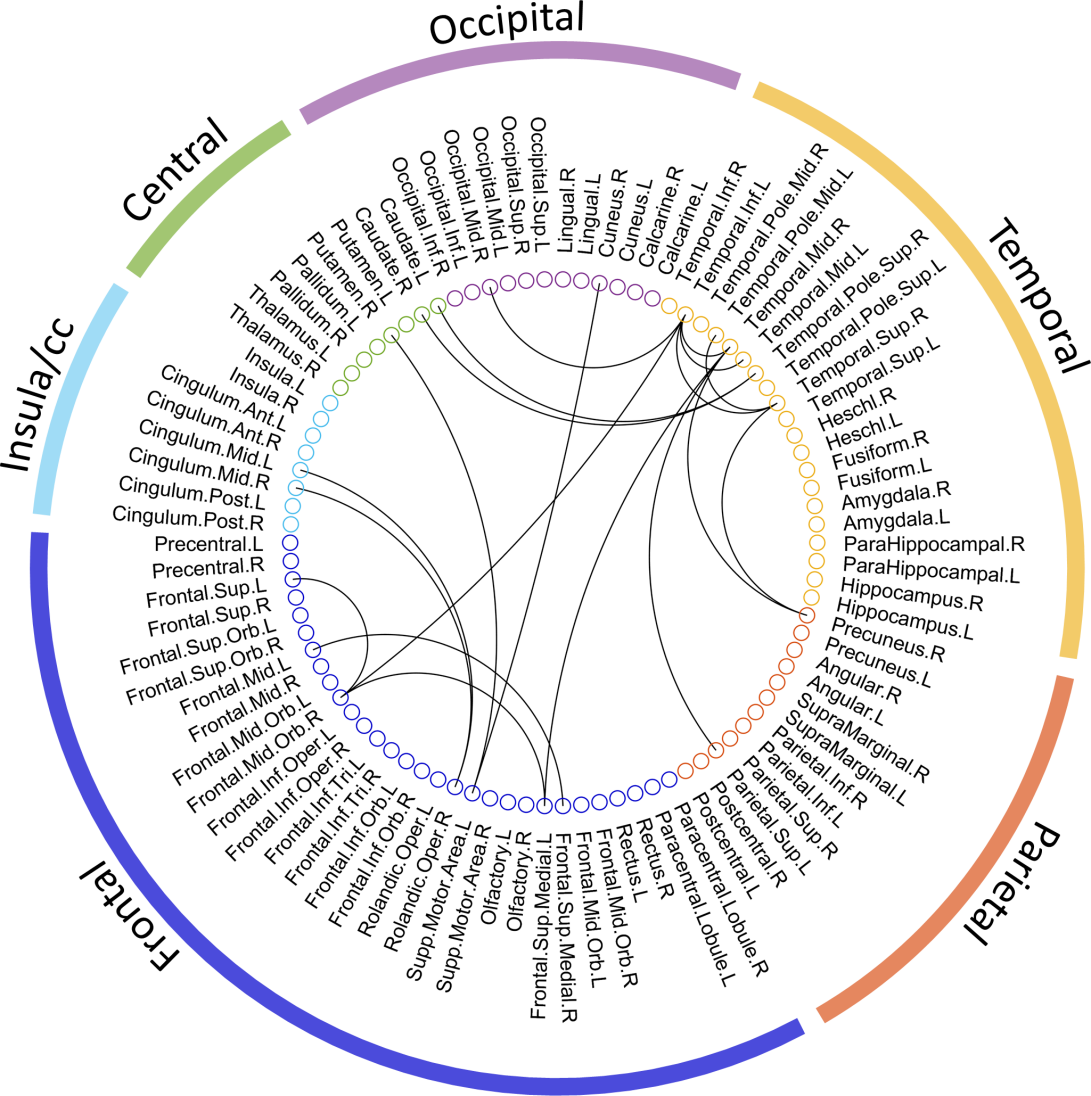
The significant metabolic connections between healthy and advanced NSCLC groups. 90 AAL ROIs derived from the 6 brain regions are presented by the colored circles under the corresponding brain regions (external colored wedge). A total of 19 significant metabolic connections are showed by the black lines.

**Figure 3:**
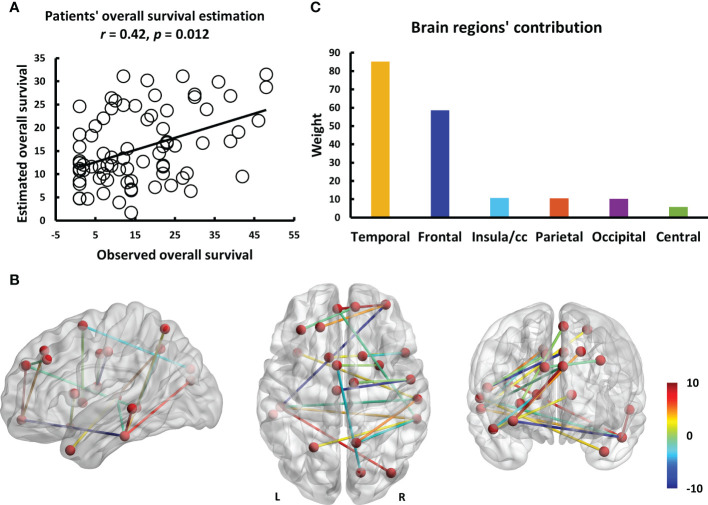
The results of estimating the overall survival in patients using individualized brain metabolic network. **(A)** The scatterplot demonstrates the significant correlation (*r* = 0.42, *p* = 0.012) between the overall survival of patients predicted by brain metabolic connectome and the actually observed overall survival. **(B)** The weight of each significant connection contributing to predicting the overall survival were mapped on the ICBM 152 template. Color bar denotes the value of connection weight. **(C)** The respective contributions of 6 brain regions.

The entire data processing procedure is shown in **Figure 1**. The connectograms in **Figure 2** depicting significant connections between healthy participants and patients were constructed using CircularGraph, which is shared by Paul Kassebaumb (http://www.mathworks.com-/matlabcentral/fileexchange/48576-circulargraph). The connection results in **Figure 3** for estimating overall survival in patients were mapped onto the International Consortium for Brain Mapping 152 template using the BrainNet Viewer software package (https://www.nitrc.org/projects/bnv).

## Results

### Clinical characteristics

In total, 78 patients and 60 healthy individuals were included in the study. There were no significant differences between the groups in terms of age and sex (*p* > 0.05), as shown in **Table 1**. The patients were followed up until March 1, 2022, during the median follow-up of 15.4 (range, 1-48) months.

**Table 1:** Clinical information of patients with advanced NSCLC group and control group.

Clinical information of NSCLCgroup and NCgroup			
	Patient	Healthy control	*p*
Age, mean(SD),y	63.9(10.05)	61.33(10.28)	0.12
male, No.(%)	65	66	0.87
Histologic subtype			
Adenocarcinoma	23	NA	
Squamous carcinoma	53	NA	
Other	3	NA	

NA, not applicable.

### Global and local graph metrics of the metabolic brain connectome

The global graph metrics of the patients are listed in **Table 2**. Ar, Q, Eglobal, Elocal, and σ increased, whereas Sr, Cp, γ, λ, Hr, and Lp decreased in the NSCLC group. Statistical analyses revealed no significant differences between the NSCLC and control groups.

**Table 2:** Global and local graph metrics of the metabolic brain connectome.

Global graph metrics	NSCLCs,mean(SD)	controls, mean(SD)	*t*	*p*	Description
*A_r_*	7.30(0.95)	7.24(0.79)	0.39	0.70	zscore
*Q*	14.21(1.36)	14.28(1.13)	-0.34	0.73	rawdata
*H_r_*	-0.68(0.35)	-0.68(0.32)	-0.02	0.99	zscore
*E_global_*	0.18(0.01)	0.17(0.01)	0.93	0.36	rawdata
*E_local_*	0.28(0.01)	0.28(0.01)	0.17	0.87	rawdata
*C_p_*	0.24(0.01)	0.24(0.01)	-0.10	0.92	rawdata
*γ*	0.88(0.09)	0.87(0.07)	0.44	0.66	rawdata
*λ*	0.60(0.01)	0.60(0.02)	-0.35	0.73	rawdata
*σ*	0.67(0.07)	0.66(0.06)	0.41	0.68	rawdata
*L_p_*	1.51(0.06)	1.52(0.08)	-0.56	0.57	rawdata
*S_r_*	-0.96(0.59)	-0.83(0.54)	-1.32	0.19	zscore

Ar, assortativity; Q, modularity score; Hr, hierarchy; Eglobal, global efficiency; Elocal, local efficiency; Cp, clustering coefficient; γ, normalized clustering coefficient; λ, normalized characteristic path length; σ, small-world;Lp, characteristic path length; Q, modularity score; Sr, synchronization.

### Degree analysis of the metabolic brain connectome

To investigate the degree distribution of the estimated metabolic brain connectome, we analyzed the mean degree of each node in the NSCLC and control groups. The degree in the Frontal.Sup.R, Frontal.Mid., Rolandic.Oper., Cingulum.Post.L, Amygdala.R, Angular., Precuneus, and Temporal.Pole.Sup.R tended to increase in the NCSLC group, whereas the degree in the Frontal.Sup.Medial.L, Frontal.Mid.Orb., Rectus.R, Insula., Cingulum.Ant.L Cuneus.R, and Temporal.Inf.L tended to decrease. The 15 significant nodes with average degrees in the NSCLC and control groups are listed in **Table 3**.

**Table 3:** Significant nodes with degree (NSCLCs vs. Controls).

Degree	*t*	*p*	Label Index
Frontal.Sup.R	2.58	0.01	4
Frontal.Mid.L	2.42	0.02	7
Rolandic.Oper.R	2.06	0.04	18
Frontal.Sup.Medial.L	-3.53	0.00	23
Frontal.Mid.Orb.R	-2.65	0.01	26
Rectus.R	-2.17	0.03	28
Insula.L	-2.05	0.04	29
Cingulum.Ant.L	-2.53	0.01	31
Cingulum.Post.L	2.02	0.05	35
Amygdala.R	2.27	0.02	42
Cuneus.R	-2.22	0.03	46
Angular.L	2.08	0.04	65
Precuneus.R	2.69	0.01	68
Temporal.Pole.Sup.R	2.22	0.03	84
Temporal.Inf.L	-3.45	0.00	89

### Betweenness analysis of the metabolic brain connectome

We also investigated the betweenness distribution of the estimated metabolic brain connectome in the NSCLC and HC groups. The results showed that betweenness in Frontal.Inf.Orb.R, Rolandic.Oper.R, ParaHippocampal.R, Amygdala.R, Fusiform.R, Precuneus.R, Thalamus.L, and Temporal.Pole.Sup.R. tended to increase in the NSCLC group, whereas betweenness in Rectus.R tended to decrease. The significant nodes with average betweenness in the healthy and advanced NSCLC groups are shown in **Table 4**.

**Table 4:** Significant nodes with betweenness (NSCLCs vs. Controls).

Betweenness	*t*	*p*	Label Index
Frontal.Inf.Orb.R	2.02	0.05	16
Rolandic.Oper.R	2.16	0.03	18
Rectus.R	-2.20	0.03	28
ParaHippocampal.R	2.73	0.01	40
Amygdala.R	2.11	0.04	42
Fusiform.R	2.93	0.00	56
Precuneus.R	2.47	0.01	68
Thalamus.L	2.12	0.04	77
Temporal.Pole.Sup.R	4.75	<0.0001	84

### Significant connections and prediction results

To explore the connections exhibited significant difference between patients and healthy participants, we reported the statistical connection analysis results. A total of 19 metabolic connections reached a significant level, as shown in **Figure 2**. Then, to evaluate whether individualized brain metabolic connectome can track patients’ overall survival, SVR model was trained to predict the overall survival for each patient. The predicted and observed overall survival in patients showed a significant correlation (*r* = 0.42, *p* = 0.012, **Figure 3A**). The raw predicted weights of each connection are shown in **Figure 3B** and listed in **Table 5**. Furthermore, grouping the connections’ predicted weights into the 6 brain regions, the connections that contributed to the overall survival prediction were predominantly located in temporal and frontal regions, as shown in **Figure 3C**.

**Table 5:** Significant connection (NSCLCs vs. Controls) and raw predicted weight.

Significant connection	Raw weight
Frontal.Mid.Orb.R - Frontal.Sup.L	4.60
Frontal.Sup.Medial.R - Frontal.Mid.L	-0.94
Frontal.Sup.Medial.L - Frontal.Mid.Orb.R	9.28
Temporal.Inf.L - Frontal.Mid.Orb.R	-13.70
Cingulum.Mid.L - Rolandic.Oper.R	-10.59
Cingulum.Mid.R - Rolandic.Oper.R	0.08
Cuneus.R - Supp.Motor.Area.L	-3.12
Putamen.R - Supp.Motor.Area.L	0.35
Temporal.Mid.R - Frontal.Sup.Medial.L	-1.16
Temporal.Inf.L - Occipital.Mid.R	7.17
Temporal.Mid.R - Parietal.Sup.L	2.77
Temporal.Sup.R - Precuneus.R	5.23
Temporal.Mid.R - Precuneus.R	-2.50
Temporal.Pole.Sup.R - Caudate.L	2.44
Temporal.Pole.Sup.R - Caudate.R	-3.06
Temporal.Pole.Mid.L - Temporal.Sup.R	2.98
Temporal.Inf.L - Temporal.Sup.R	-1.50
Temporal.Inf.L - Temporal.Mid.L	15.04
Temporal.Inf.L - Temporal.Mid.R	4.05

## Discussion

We present an individualized metabolic network using FDG-PET imaging in patients with advanced NSCLC. These images were feeded to a machine learning model, which was then used to predict patients’ overall survival. Nervous system-cancer crosstalk is bidirectional and is called “cancer neuroscience” (4, 34). Evidence has demonstrated that diverse cancers may elicit specific functional networks of interconnected brain regions, resulting in specific structural and metabolic changes in the brain (10, 35, 36). Studies have shown that metabolism in the brain is coupled to synaptic activity in a putative association; therefore, metabolic changes in the brain reflect changes in synaptic activity to a certain extent (37, 38). The characteristics of the metabolic brain network obtained by the KLS method may be related to synapses and neural activities as well as the psychology of patients and cancer. We further used the machine learning model obtained by the brain metabolic network as a feature to predict the overall survival and actual survival of patients with NSCLC to achieve a good fit. The lung-brain axis is a cutting-edge area of research (39), as studies have shown that the microbial community in the lungs can impact metabolic and structural changes in the brain, leading to brain-related immune diseases (40, 41). Our research aims to construct a model by collecting FDG PET metabolic information from a small sample of late-stage non-small cell lung cancer patients and linking it to the patients’ overall survival. We hope that this model will eventually provide a potential biomarker for clinical decision-making in regards to a patient’s overall survival, but this still requires further support from additional samples.

We found that the NSCLC group had no statistical difference in global and local graph metrics than that of the normal group; however, Ar, Q, Eglobal, Elocal, Cp, γ, λ, σ, and Sr increased, whereas Sr,Cp, γ, λ, Hr, and Lp decreased in the NSCLC group. This result is interesting and differs from those of previous research. Previous FDG-PET imaging studies involving patients with no-CNS tumor metabolic networks may have used group-level analyses, which potentially sacrifice or obscure salient individual differences within a group; in contrast, our novel KLS approach offers construction of an individual’s metabolic brain network, which varies from most FDG PET and MR studies (42–44).

Based on the novel KLS approach, we were able to parameterize the balance between short- and long-range functional connections, we found several abonaml nodes in the degree analysis and betweenness analysis. We also found that the frontal and temporal lobes made up a larger portion both in degree and betweenness analyses. Abnormal metabolism in the prefrontal cortex is associated with aggression and impulsivity, which are prevalent in patients with cancer (45). A previous study on brain metabolism and depression in no-CNS cancers also found that abnormal frontal lobe metabolism was correlated with depression (44). Furthermore, these regions may be related to the severity of depression (46). Research has shown that temporal brain regions are involved in emotional processing and declarative memory (47). Similarly, in previous studies, structural and functional abnormalities were found in the temporal lobe (10, 35, 48). Based on previous findings, temporal and frontal lobe abnormalities are often concomitant in patients with no-CNS tumors, which were also observed in our study. We speculate that this frontotemporal lobe abnormality is likely to be associated with the default mode network, leading to emotional disturbance and cognitive deficits in patients with advanced NSCLC (49–51).

We also found abnormalities in the limbic system (parahippocampal gyrus, thalamus, amygdala, and cingulate gyrus) in the brain metabolic networks. The limbic system is involved in mediating instinctual and affective behaviors, consolidating memories, and forming emotions (52, 53). We focused on metabolic abnormalities in the amygdala because, in addition to affecting emotional processing, it has recently been implicated in nociceptive processing (54). We assumed that an abnormal metabolism network in the amygdala may have a connection with the cancerous pain experienced by patients with advanced NSCLC. Further research is required to confirm this hypothesis.

In addition to betweenness and degree analyses, we explored abnormal metabolic brain network connections in patients with NSCLC and used these abnormal connections to develop a prediction model. Because our present brain connectome approach can measure local network properties and the global network, it could powerfully identify salient properties predictive of the overall survival of patients with advanced NSCLC. We found that our model had a good fit with the observed overall survival (r=0.42, *p*=0.012). The connections with larger contributions are Temporal.Inf.L - Temporal.Mid.L, Temporal.Inf.L - Frontal.Mid.Orb.R, Cingulum.Mid.L - Rolandic.Oper.R, and Frontal.Sup.Medial.L - Frontal.Mid.Orb.R. We found that the temporal and frontal lobes play a large role in the prediction, and fronto-temporal function is a characteristic alteration of the metabolic brain network in patients with advanced NSCLC, which is associated with memory, emotion, and cognitive changes. Cingulum.Mid.L - Rolandic.Oper.R abnormalities were not mentioned in previous studies, and abnormalities in this connection are associated with cognition and are frequently observed in schizophrenia (55). Tumors may affect brain function through a variety of factors and cannot be fully explained at present. Similarly, the machine learning prediction model had a good degree of fit with the actual survival period; however, further research is needed for a better understanding and usage of the model.

Presently, functional magnetic resonance imaging (fMRI) is also a widely adopted method in the study of cerebral glucose metabolism (56, 57). Presently, functional magnetic resonance imaging (fMRI) is also a widely adopted method in the study of cerebral glucose metabolism. This presents new opportunities for future investigations, and highlights the potential for incorporating multiple imaging modalities to enhance the study of brain metabolism.

This retrospective study had few limitations. We were unable to systematically assess various brain functions such as cognition and memory in patients, which we aim to include in future studies. Our study had a small sample size; we hope to expand the sample size in the follow-up studies. In addition, the healthy control group would have been a good additional reference but would have been difficult to justify. Despite having divided the pathology data of our NSCLC patients into groups based on their histologic subtypes, the insufficient number of patients in each subtype has resulted in the combination of all subtypes into a single group for this study. In the future, we plan to continue collecting patient data and analyze it in detail based on pathology.

## Conclusion

In this study, we found that unique changes in the brain metabolic network may be closely related to patients’ mental status and cognition, which is critical to the understanding of the neurobiological mechanisms associated with depression symptoms in patients with advanced NCSLC. In addition, since our predictive model predicts overall survival with higher significance, it would largely benefit in the clinical practice.

## Data availability statement

The raw data supporting the conclusions of this article will be made available by the authors, without undue reservation.

## Ethics statement

The studies involving human participants were reviewed and approved by Medical Ethics Committee of The Fifth Affiliated Hospital of Sun Yat-sen University. Written informed consent for participation was not required for this study in accordance with the national legislation and the institutional requirements.

## Author contributions

JY: Writing original manuscript, study design. LH: Writing original manuscript, data analysis, developing model. XLC: Data collection. QLC: Data collection. XLZ: Data analysis. ZY: Quality control, study design. YW: Editing and reviewing manuscript, study design. All authors contributed to the article and approved the submitted version.
